# Adverse drug reactions in South African patients receiving bedaquiline-containing tuberculosis treatment: an evaluation of spontaneously reported cases

**DOI:** 10.1186/s12879-019-4197-7

**Published:** 2019-06-20

**Authors:** Jackie Jones, Vanessa Mudaly, Jacqueline Voget, Tracey Naledi, Gary Maartens, Karen Cohen

**Affiliations:** 1 Medicines Information Centre, Division of Clinical Pharmacology, Department of Medicine, University of Cape Town, K45, Old Main Building, Groote Schuur Hospital, Observatory, Cape Town, 7945 South Africa; 2 Health Programmes, Department of Health, Western Cape Government 1st Floor, Norton Rose House, 8 Riebeek Street, Cape Town, 8000 South Africa; 3 School of Public Health and Family Medicine, University of Cape Town, Falmouth Rd, Observatory, Cape Town, 7945 South Africa; 4 Division of Clinical Pharmacology, Department of Medicine, University of Cape Town, K45, Old Main Building, Groote Schuur Hospital, Observatory, Cape Town, 7945 South Africa

**Keywords:** Drug-resistant TB, Pharmacovigilance, Drug safety, Bedaquiline

## Abstract

**Background:**

Bedaquiline was recently introduced into World Health Organization (WHO)-recommended regimens for treatment of drug resistant tuberculosis. There is limited data on the long-term safety of bedaquiline. Because bedaquiline prolongs the QT interval, there are concerns regarding cardiovascular safety. The Western Cape Province in South Africa has an established pharmacovigilance programme: a targeted spontaneous reporting system which solicits reports of suspected adverse drug reactions (ADRs) in patients with HIV-1 and/or tuberculosis infection. Since 2015, bedaquiline has been included in the treatment regimens recommended for resistant tuberculosis in South Africa. We describe ADRs in patients on bedaquiline-containing tuberculosis treatment that were reported to the Western Cape Pharmacovigilance programme.

**Methods:**

We reviewed reports of suspected ADRs and deaths received between March 2015 and June 2016 involving patients receiving bedaquiline-containing tuberculosis treatment. A multidisciplinary panel assessed causality, and categorised suspected ADRs using World Health Organisation-Uppsala Monitoring Centre system categories. “Confirmed ADRs” included all ADRs categorised as definite, probable or possible. Preventability was assessed using Schumock and Thornton criteria. Where a confirmed ADR occurred in a patient who died, the panel categorised the extent to which the ADR contributed to the patient’s death as follows: major contributor, contributor or non-contributor.

**Results:**

Thirty-five suspected ADRs were reported in 32 patients, including 13 deaths. There were 30 confirmed ADRs, of which 23 were classified as “possible” and seven as “probable”. Bedaquiline was implicated in 22 confirmed ADRs in 22 patients. The most common confirmed ADR in patients receiving bedaquiline was QT prolongation (8 cases, 7 of which were severe). A fatal arrhythmia was suspected in 4 sudden deaths. These 4 patients were all taking bedaquiline together with other QT-prolonging drugs. There were 8 non-bedaquiline-associated ADRs, of which 7 contributed to deaths.

**Conclusions:**

Confirmed ADRs in patients receiving bedaquiline reflect the known safety profile of bedaquiline. Quantifying the incidence and clinical consequences of severe QT-prolongation in patients receiving bedaquiline-containing regimens is a research priority to inform recommendations for patient monitoring in treatment programmes for drug resistant tuberculosis. Pharmacovigilance systems within tuberculosis treatment programmes should be supported and encouraged, to provide ongoing monitoring of treatment-limiting drug toxicity.

## Background

South Africa has high tuberculosis (TB) incidence and a growing epidemic of multidrug resistant (MDR) and extensively drug resistant (XDR) TB [1]. Drug resistant TB treatment regimens are prolonged, poorly tolerated, and result in poor outcomes [2]. The oral diarylquinoline, bedaquiline, was approved by the Food and Drug Administration (FDA) for the treatment of MDR-TB in 2012, under the provisions of the accelerated approval regulations for serious or life-threatening illnesses [3]. This approval was based on the results of two phase IIb clinical trials in which patients with MDR- TB were randomised to bedaquiline or placebo, in combination with a five drug background regimen: bedaquiline significantly reduced time to sputum culture conversion and increased the proportion with culture conversion [4, 5]. In 2013, WHO recommended that bedaquiline be used in treatment of MDR and XDR TB, but emphasized the need for robust pharmacovigilance strategies in TB treatment programmes to actively monitor safety of bedaquiline [6].

Two potentially fatal adverse drug reactions (ADRs) from bedaquiline are QT prolongation and liver injury. Greater QT prolongation was seen in the bedaquiline arm of the phase 2 trial (mean increase in QT 15.4 ms with bedaquiline versus 3.3 ms with placebo). In the trial there was also unexplained excess mortality in bedaquiline-exposed participants [5]. Almost all of the deaths in the bedaquiline arm occurred after the bedaquiline was stopped, but bedaquiline has a very long half-life (5.5 months) [7] and could therefore still be implicated in these deaths.

Bedaquiline was officially introduced into the TB treatment programme in South Africa in March 2015, for use in selected patients with MDR (either drug intolerance or both *inhA* and *katG* mutations) and in all patients with pre-XDR and XDR-TB [8]. Western Cape Department of Health treatment guidelines recommended baseline and monthly ECG monitoring for the duration of bedaquiline therapy [9]. The quinolone moxifloxacin prolongs the QT interval more than levofloxacin [10] therefore the guideline recommended switching from moxifloxacin to levofloxacin to minimize additive QT prolongation. According to Western Cape Department of Health treatment guidelines, a QTc interval > 500 msec or an increase > 50 msec above baseline without symptoms or the presence of Torsades de pointes or other serious ventricular dysrhythmias was graded as severe, with bedaquiline withdrawal advised [9].

When bedaquiline was introduced into the TB treatment programme, health care workers in the Western Cape Province of South Africa were actively encouraged to report all suspected serious adverse drug reactions and all deaths in patients receiving bedaquiline-containing TB treatment to the Western Cape Pharmacovigilance Programme (a targeted spontaneous reporting system for suspected ADRs in patients with HIV and/or TB infection). We describe these reports.

## Methods

The Western Cape provincial HIV and TB programmatic pharmacovigilance system, founded in 2005, is a collaboration between the Western Cape Province Department of Health and the Division of Clinical Pharmacology at the University of Cape Town. The pharmacovigilance programme solicits reports of suspected ADRs that are life-threatening, result in hospitalisation/prolonged hospitalisation, result in permanent disability or incapacity, or require cessation of drug, as well as reports of deaths and birth defects, from all public health sector facilities in the Western Cape Province. Reports are submitted on a structured reporting form which requests the following information (if applicable): patient age, sex, weight, pregnancy status, HIV status, CD4 count, co-morbidities, medication history (including all concomitant medications), details of the adverse event, relevant laboratory investigations and management of the adverse event, including response to drug withdrawal if this occurs. Reports are prospectively entered into a repository. A pharmacist performing initial assessment of the report contacts the reporter to request additional clinical information for causality and preventability assessment, if required.

During the first year after bedaquiline was introduced into the TB treatment programme, reporting of suspected ADRs was actively encouraged by the provincial department of health, with repeated reminders to clinicians treating resistant TB. We describe suspected ADRs and deaths in patients on bedaquiline reported to the pharmacovigilance system from March 2015, when bedaquiline was introduced into the programme, until June 2016.

Causality assessment of suspected ADRs was performed using the World Health Organisation-Uppsala Monitoring Centre (WHO-UMC) system [11]. Causality assessment was performed by a team comprising an information pharmacist, a clinical pharmacologist and a physician, with input from other specialties, if required. “Confirmed ADRs” included all suspected ADRS that we categorised as definite, possible or probable according to the WHO-UMC criteria [11]. A definite ADR required: an event or laboratory abnormality with a plausible temporal association with the drug, can’t be explained by the disease or other drugs, plausible response to withdrawal, a pharmacologically or phenomenologically definitive event and a positive rechallenge (if indicated). A probable ADR required: an event or laboratory abnormality with a plausible temporal association with the drug, unlikely to be due to disease or other drugs, response to withdrawal reasonable and rechallenge not a prerequisite. A possible ADR required: an event or laboratory abnormality with a plausible temporal association with the drug, could be explained by disease or other drugs and information on drug withdrawal is lacking/unclear [11]. In the event of death, we further categorised the ADR as a contributor, major contributor or non-contributor to the patient’s death.

Preventability was assessed using Schumock and Thornton criteria [12]; if any of these criteria were met the ADR was categorised as preventable. Criteria are as follows: drug prescribed inappropriate, dose/route/frequency inappropriate, required laboratory monitoring not performed, history of a previous reaction to the drug, drug interaction, toxic serum drug level and poor compliance.

Continuous variables were summarised as mean and standard deviation (SD) if normally distributed, and median and interquartile range (IQR) if non-parametrically distributed.

## Results

We received reports of suspected adverse drug reactions (ADRs) involving 32 patients on bedaquiline-containing TB therapy between March 2015 and June 2016. These included 13 reports of deaths. During this time, 549 patients were initiated on bedaquiline in the Western Cape.

The reports came from urban healthcare facilities managing resistant TB, and all but one were submitted by doctors (31/32, 97%). Patient characteristics are described in Table 1. Half of the reports involved patients known to be infected with HIV, with a median CD4 count of 51 cells/μL (IQR 37 to 65). HIV status was missing in seven cases (22%).

**Table 1:** Patient characteristics (*n* = 32)

Characteristic	
Median age (IQR) years	30 (21 to 37)
Male sex [n (%)]	12 (38%)
Known HIV- infected [n (%)]	16 (50%)
Mean number of concomitant medicines (SD)	7.4 (1.5)
Median days on bedaquiline (IQR)	38.5 (13.5 to 88)

Thirty-five suspected ADRs were reported in the 32 patients. We confirmed 30 ADRs, of which we assessed 23 ADRs as “possible” and seven as “probable”. The 30 confirmed ADRs are described in Table 2. The most commonly reported ADR was QTcF prolongation in 8 patients. Seven of these patients had severe QTcF prolongation. Bedaquiline was implicated in 22 of the confirmed ADRs (73%); in all of these ADRs there were one or more concomitant drugs that were also implicated. In eight of the confirmed ADRs (27%) the causality assessment panel attributed causality to a drug/drugs other than bedaquiline. Seven of these non-bedaquiline-associated ADRs contributed to deaths.

**Table 2:** Confirmed ADRs

Organ system	ADR	Number of reports	Number of patients who died in whom ADR occurred (number of deaths in which ADR was implicated)	Implicated drugs^a^ (number of reports in which drug implicated)
Cardiovascular	Sudden death -suspected arrhythmia	4	4 (4)	Bedaquiline (4)
				Clofazimine (4)
				Levofloxacin (4)
	QTcF prolongation	8	1 (0)	Bedaquiline (8)
				Clofazimine (7)
				Levofloxacin (6)
				Moxifloxacin (1)
Skin	Acneiform rash	2	0	Bedaquiline (2)
				Clofazimine (2)
				Emtricitabine (1)
				Ethionamide (1)
				Isoniazid (1)
				Levofloxacin (2)
				Linezolid (2)
				Lopinavir/ritonavir (1)
				Para-aminosalicylic acid (2)
				Pyrazinamide (2)
				Tenofovir (1)
				Terizidone (2)
	Itchy rash	1	0	Bedaquiline (1)
				Clofazimine (1)
				Linezolid (1)
				Para-aminosalicylic acid (1)
	Pustular facial rash	3	0	Bedaquiline (3)
				Clofazimine (3)
				Ethionamide (1)
				Isoniazid (1)
				Lamivudine (1)
				Levofloxacin (3)
				Linezolid (3)
				Nevirapine (1)
				Para-aminosalicylic acid (3)
				Pyrazinamide (3)
				Tenofovir (1)
				Terizidone (3)
	Rash	1	0	Bedaquiline (1)
				Clofazimine (1)
				Isoniazid (1)
				Levofloxacin (1)
				Linezolid (1)
				Para-aminosalicylic acid (1)
				Pyrazinamide (1)
				Terizidone (1)
Gastrointestinal	Antibiotic-associated diarrhoea	2	2 (2)	Levofloxacin (1)
				Moxifloxacin (1)
Musculoskeletal	Arthralgia	3	0	Bedaquiline (3)
				Levofloxacin (1)
				Para-aminosalicylic acid (3)
				Pyrazinamide (2)
Haematological	Increased bleeding risk	1	1 (1)	Warfarin (1)
	Pancytopenia	1	1 (1)	Linezolid (1)
Neurologic	Psychosis	1	0	Levofloxacin (1)
				Terizidone (1)
	Seizure	1	1 (1)	Moxifloxacin (1)
Renal	Renal failure	2	2 (2)	Kanamycin (2)

^a^ Multiple drug suspects may be implicated in an ADR

In nine of the 13 deaths we identified one or more ADRs that contributed to the death (Table 2). Three deaths were assessed as “disease-related” and one was “unassessable” due to the paucity of information provided by the reporter. There were four sudden deaths due to suspected arrhythmia. In all of these cases, patients were on bedaquiline in combination with other drugs known to cause QT prolongation (see Table 2). In addition, one of these patients had a history of hypokalaemia and hypomagnesaemia. The other ADRs implicated in deaths were antibiotic-associated diarrhoea (moxifloxacin implicated), pancytopenia (linezolid implicated), and elevated INR with increased bleeding (warfarin implicated). There was more than one ADR implicated in two deaths. The first of these patients had antibiotic-associated diarrhoea with confirmed *C. difficile* on stool culture (levofloxacin implicated) and renal failure (kanamycin implicated). The second patient had renal failure (kanamycin implicated) and a seizure (moxifloxacin implicated).

We received five reports of pustular/acneiform rashes. All of these patients were taking multiple concomitant medications that could be implicated, making causality attribution difficult. None of the rashes were treatment-limiting or life threatening.

Two preventability issues were identified. One patient remained on moxifloxacin after starting bedaquiline instead of being switched to levofloxacin. Levofloxacin is the preferred quinolone to use in combination with bedaquiline as it has a lesser effect on QT prolongation. A second patient had a raised INR whilst on warfarin treatment and no adjustment in warfarin dose was recorded on the report.

## Discussion

The ADRs reported to the Western Cape pharmacovigilance programme are consistent with the known safety profile of bedaquiline and other drugs used in drug resistant TB treatment. The most commonly reported ADR in our case series was QTcF prolongation. In all cases patients were receiving multiple QT-prolonging agents: bedaquiline in combination with clofazamine and/or either moxifloxacin or levofloxacin. The four sudden deaths were all in patients receiving multiple QT-prolonging drugs.

Rashes, in particular pustular/acneiform rashes, were commonly reported. All patients were on multiple medicines that could be implicated. None of the rashes were severe.

Previous studies have reported a similar pattern of ADRs attributed to bedaquiline. In a phase 2 randomised controlled trial of bedaquiline versus placebo (on a background of 5 additional anti-tuberculosis drugs) there was an increased risk of QTcF prolongation with bedaquiline [5]. There was also an unexplained increased risk of death associated with bedaquiline in this study- 10 out of 79 patients randomised to bedaquiline and 2 out of 81 randomised to placebo died [4, 5]. In an open-label, single arm observational study 2 out of 233 patients receiving bedaquiline developed clinically significant QTcF prolongation (> 500 msec)- both patients were also receiving clofazimine concomitantly [13]. These studies prompted the inclusion of a black box FDA warning of increased risk of sudden death and QTcF prolongation in the prescribing information [7]. Prescribers were advised to prescribe bedaquiline only if alternative effective treatment is not available, and cautioned about prescribing concomitant drugs known to have effects on the QT interval, which may have additive effects [7]. However, in practice there is a limited number of drugs known to be effective against MDR-TB, and many of these affect the QT interval (bedaquiline, clofazimine, moxifloxacin and to a lesser extent, levofloxacin).

There is limited data on cardiovascular safety of long-term bedaquiline exposure. A South African observational cohort reported minimal increase of QT after bedaquiline initiation, but this study is flawed in that a third of the patients were taking moxifloxacin, which is known to prolong QT, at the time of baseline ECG [8]. In 24 patients in that cohort, QT increased by more than 50 ms after bedaquiline initiation, with QT > 500 ms in three patients [8]. QTcF> 500 ms occurred in 11% of patients treated with bedaquiline in a French observational cohort but no arrhythmias were reported [14]. A systematic review of the available evidence on the cardiac safety of bedaquiline reported QTc prolongation > 500 msec in 3.2% of patients [15].

QT prolongation was the most frequently reported ADR in our case series. QT prolongation was reported in 1.4% of patients initiated on bedaquiline-containing TB treatment during the study period (8/549); severe QTc prolongation was reported in 1.2% (7/549). The four sudden deaths reported are concerning, as all were in patients receiving three drugs known to prolong the QT. Current treatment protocols recommend baseline and monthly ECGs for patients receiving bedaquiline. Access to ECG monitoring in resource limited settings is limited. However, more data on long term cardiovascular safety of bedaquiline-containing regimens, and incidence of life-threatening arrhythmias is required to guide future recommendations for baseline and on-treatment ECG monitoring. This is of particular importance as MDR and XDR treatment regimens frequently include multiple QT- interval-prolonging drugs.

A recent large retrospective cohort study in South Africa compared outcomes in patients with resistant TB treated with bedaquiline containing regimens to those not receiving bedaquiline and found that bedaquiline improved survival in both MDR and XDR TB [16]. These data are reassuring, especially in view of the black box warning about bedaquiline potentially increasing mortality. As use of bedaquiline expands in TB treatment programmes, pragmatic guidance regarding cardiovascular monitoring is needed.

This study has several limitations. Cases were reported to a voluntary, targeted spontaneous reporting system. There is likely to be under-reporting, and we cannot calculate incidence. Causality assessment was challenging as patients were often extremely ill and on multiple medications. A strength of our approach is that causality assessment was performed by a multidisciplinary team.

## Conclusion

MDR-TB and especially XDR-TB has a poor prognosis, and bedaquiline-containing therapy may improve cure rates and reduce mortality [8, 16]. However, the drugs used in MDR treatment have significant associated toxicities. Pharmacovigilance systems should be supported and encouraged within MDR-treatment programmes, to provide ongoing monitoring for treatment-limiting toxicity. Robust data on incidence and prevalence of serious ADRs in patients receiving bedaquiline containing TB treatment, and QT prolongation in particular, is a research priority to guide programmatic recommendations for optimal monitoring of patients receiving bedaquiline.

## Data Availability

The datasets used and/or analysed during the current study are available from the corresponding author on reasonable request.

## Abbreviations

ADRs: Adverse drug reactions
ECG: Electrocardiogram
FDA: Food and Drug Administration
HIV: Human immunodeficiency virus
INR: International normalised ratio
MDR: Multi-drug resistant
QTcF: QT interval corrected by Fredericia’s formula
TB: Tuberculosis
WHO: World Health Organisation
XDR: Extensively drug resistant
